# Stress-related changes in leukocyte profiles and telomere shortening in the shortest-lived tetrapod, *Furcifer labordi*

**DOI:** 10.1186/s12862-020-01724-2

**Published:** 2020-12-01

**Authors:** Falk Eckhardt, Angela Pauliny, Nicky Rollings, Frank Mutschmann, Mats Olsson, Cornelia Kraus, Peter M. Kappeler

**Affiliations:** 1 Department Sociobiology/Anthropology, Institute of Zoology and Anthropology, University of Göttingen, Kellnerweg 6, 37077 Göttingen, Germany; 2 Department of Biological and Environmental Science, University of Gothenburg, Medicinaregatan 18A, 41390 Göteborg, Sweden; 3 School of Life and Environmental Sciences, University of Sydney, Sydney, NSW Australia; 4 Exomed-Labor, Schönhauser Straße 62, 13127 Berlin, Germany; 5 Behavioral Ecology and Sociobiology Unit, German Primate Center, Leibniz Institute of Primatology, Kellnerweg 4, 37077 Göttingen, Germany

**Keywords:** *Furcifer labordi*, Life history, Telomeres, H/L ratio, Body condition

## Abstract

**Background:**

Life history theory predicts that during the lifespan of an organism, resources are allocated to either growth, somatic maintenance or reproduction. Resource allocation trade-offs determine the evolution and ecology of different life history strategies and define an organisms’ position along a fast–slow continuum in interspecific comparisons. Labord’s chameleon (*Furcifer labordi*) from the seasonal dry forests of Madagascar is the tetrapod species with the shortest reported lifespan (4–9 months). Previous investigations revealed that their lifespan is to some degree dependent on environmental factors, such as the amount of rainfall and the length of the vegetation period. However, the intrinsic mechanisms shaping such a fast life history remain unknown. Environmental stressors are known to increase the secretion of glucocorticoids in other vertebrates, which, in turn, can shorten telomeres via oxidative stress. To investigate to what extent age-related changes in these molecular and cellular mechanisms contribute to the relatively short lifetime of *F. labordi*, we assessed the effects of stressors indirectly via leukocyte profiles (H/L ratio) and quantified relative telomere length from blood samples in a wild population in Kirindy Forest. We compared our findings with the sympatric, but longer-lived sister species *F.* cf. *nicosiai,* which exhibit the same annual timing of reproductive events, and with wild-caught *F. labordi* that were singly housed under ambient conditions.

**Results:**

We found that H/L ratios were consistently higher in wild *F. labordi* compared to *F.* cf. *nicosiai*. Moreover, *F. labordi* already exhibited relatively short telomeres during the mating season when they were 3–4 months old, and telomeres further shortened during their post-reproductive lives. At the beginning of their active season, telomere length was relatively longer in *F.* cf. *nicosiai*, but undergoing rapid shortening towards the southern winter, when both species gradually die off. Captive *F. labordi* showed comparatively longer lifespans and lower H/L ratios than their wild counterparts.

**Conclusion:**

We suggest that environmental stress and the corresponding accelerated telomere attrition have profound effects on the lifespan of *F. labordi* in the wild, and identify physiological mechanisms potentially driving their relatively early senescence and mortality.

## Background

Life history theory is based on the premise that during the lifetime of an individual, energy and resources are allocated to either growth, somatic maintenance, or reproduction [1–3]. For example, resources, which are invested into fast growth and early reproduction, cannot be used for somatic maintenance, which may lead to shorter lifespans. The trade-offs between traits shape life history strategies and the distribution of species along a fast–slow continuum of life history speeds [4–6]. In spite of the supposed significance of extrinsic factors in shaping life histories, aging research is still largely biased towards captive animals living under standardized, optimal conditions (e.g., [7]). In the wild, studies of senescence have largely focused on long-lived animals that face relatively low levels of extrinsic mortality (e.g. sea turtles [8], birds [9], Soay sheep [10]). However, studies focusing on age-related changes in short-lived species in the wild are rare. Hence, studies of wild populations with high extrinsic mortality are essential for testing hypotheses on the evolution of lifespan and senescence.

Oxidative stress and its damage to macromolecules is one of the most cited causes of aging [11]. The oxidative damage is a byproduct of aerobic respiration [12] and intensified by chronic stress conditions characterized by a persistent release of glucocorticoids (GCs) in vertebrates [13]. Physiological stress is an important mediator in the trade-off between survival and reproduction [14, 15]. GCs are released in response to a wide range of stressful stimuli (e.g., [16]), and several of their effects parallel those observed during aging, suggesting that chronic stress has a potential to accelerate the aging process [17, 18].

The immunosuppressive effects of chronic GC elevation and their consequences for morbidity and mortality have been studied intensively [19, 20]. Alterations in key immunological parameters during chronic stress parallel those during normal immunosenescence to a large degree [21]. These hormones are important regulators of carbohydrate, lipid, and protein metabolism [22], and several earlier studies linked poor body condition to elevated GC concentrations (e.g., [23]). The direct measurement of baseline GC levels in wildlife via blood plasma can be challenging as stress hormones can rise immediately following capture [24]. However, leukocyte profiles are a suitable tool to indirectly assess stress levels as these hormones increase the number of heterophils and decrease the number of lymphocytes. Leukocyte responses to stress take about 12 h to several days in ectotherms (reviewed in [25]). Heterophils are the primary phagocytic leukocyte, which proliferate in circulation in response to infections, inflammation and stress [26–30]. Lymphocytes are involved in a variety of immunological functions such as the production of immunoglobulin and modulation of immune defense [31].

At the cellular level, telomere length (TL) and shortening are thought to be significant proximate contributors to the aging process. Telomeres are short, tandem-repeated sequences of DNA found at the ends of linear eukaryotic chromosomes, whose sequence (TTAGGG) is highly conserved among vertebrates [32]. Telomeres function in stabilizing chromosomal end integrity [33], inhibiting aberrant fusions and rearrangements that occur on broken chromosomes [34], and aiding in the completion of duplication [35]. During each cell cycle, telomeric repeats are lost because DNA polymerase is unable to completely replicate the 3′end of linear DNA [35].

There is great variation among species in age-specific TL [36]. Sexual differences in TL and attrition have been suggested to contribute to sex-specific disease and mortality patterns in humans [37, 38], where women typically have longer telomeres and are longer-lived (e.g., [39]). Telomerase, the enzyme that countervails telomere shortening was found to be active in stem cells, gametes and most cancer cells, but normally absent from or at very low levels in most somatic cells [40]. However, some studies in reptiles suggested that telomerase may not be turned off in adult somatic cells [41]. Besides cell division dependent telomere shortening, elevated levels of corticosterone can further affect TL via increased oxidative damage by reactive oxygen species (ROS) [42, 43]. Elevated GCs, particularly during long-term physiological or psychological stress, have been linked to increased oxidative stress and concomitant telomere shortening and reduced telomerase activity [43, 44]. As the nucleobase guanine is a major oxidation target for ROS, the (TTAGGG) repeats are particularly exposed to oxidative damage [45].

Telomeres may also act as sentinels of the general level of DNA damage in a given cell. High levels of telomere damage would be indicative of high levels of damage to the coding sequences. Thus, telomeres could offer a mechanism to ensure that cells with high levels of DNA damage soon terminate division [46]. Overall, demanding life history stages and harsh environmental conditions seem to be linked to a rapid rate of telomere degradation, and there is also a clear connection between physiological stress and telomere attrition in humans, laboratory rodents and wild vertebrates [44, 47–50]. This evidence suggests that telomere dynamics could be closely related to stress in wild vertebrates (reviewed in [51]), and Houben et al. [52] emphasized that telomeres are a promising biomarker for chronic oxidative stress.

Labord’s chameleon (*Furcifer labordi*) from the seasonal deciduous dry forests in western and southwestern Madagascar has a lifespan of only 4–9 months [53, 54]. This extreme life history makes this species an interesting model for studying potential mechanisms of accelerated senescence, especially because longer-lived sympatric congeners are available for comparative studies. During their short lives, these chameleons hatch at the beginning of the wet season in November, passing through subsequent fast juvenile growth, maturation and courtship, followed by the death of both sexes during the early dry season in May [53, 54]. Wild females tend to live slightly longer, whereas no sex difference in lifespan was found in caged individuals kept under ambient conditions [54]. Fast growth rates, high reproductive rates and intense mating competition might proximately contribute to increased stress levels and telomere shortening, which in turn may facilitate the decrease of physiological functioning, ultimately leading to death (e.g., [55, 56]).

To investigate whether the ratio of heterophils and lymphocytes (H/L ratio) and telomere shortening are associated with the early die-off in *F. labordi* in the wild, we determined their telomere dynamics as well as their leukocyte profiles as an indict measure of physiological stress. Our study included two comparisons; one between wild *F. labordi* and their sympatric and longer-lived congener *F.* cf. *nicosiai*, and one with *F. labordi* kept in single cages under ambient conditions, shielding them substantially from environmental stressors, like hunger or predation risk. We predicted an increase in H/L ratios as well as rapidly shortening telomeres in post-reproductive wild *F. labordi* as well as lower H/L ratios and decelerated telomere attrition in *F.* cf. *nicosiai*. Furthermore, as age-related changes should be delayed in the longer-lived females of both species, we predicted females to exhibit comparatively slower rate of senescence than males. Finally, caged *F. labordi*, which were shielded from extrinsic mortality and from a substantial part of the costs of reproduction and starvation, were expected to exhibit slower correlates of aging compared to their wild conspecifics.

## Results

**Fig. 1:**
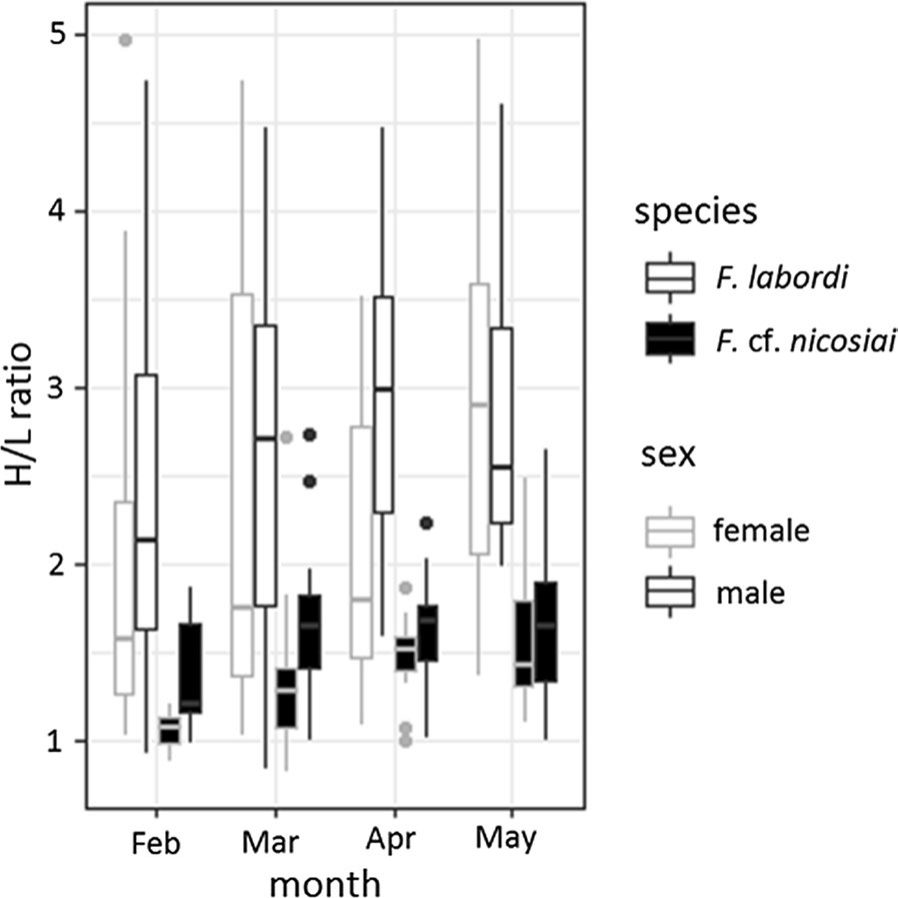
H/L ratio of adult wild *F. labordi* (n = 319) and *F.* cf. *nicosiai* (n = 103)*.* Boxplots depict H/L ratio per species and sex from February until May, covering the period between mating and death

In both species, heterophils were the most abundant leukocyte type, followed by lymphocytes azurophils and basophils. Heterophiles exhibited a spherical shape with an eccentric mostly lobed nucleus containing clumpy basophilic purplish chromatin. Most lymphocytes contained a large nucleus with coarse chromatin, leaving only a small visible band of cytoplasma around it. Basophils were only found sporadically. On average, the H/L ratio of *F. labordi* (2.45 ± 0.97 SD, n = 319) was significantly higher compared to that of *F.* cf. *nicosiai* (1.51 ± 0.47 SD, n = 103, t = − 9.921, p < 0.001). Moreover, we detected an increase of the H/L ratio in both species between February and May (Fig. 1, Table 1), reflecting the cessation of mating activities. In captive specimen, we found an average H/L profile of (1.42 ± 0.14 SD, n = 40) and no significant sex differences (Table 2). As in their wild conspecifics, the H/L ratio of captive chameleons increased significantly from February until June (Table 2).

**Table 1:** Parameters of the linear model examining the influence of time, sex and species on H/L ratio

Fixed effects	Estimate	SE	t-value	P	F	df	P
(Intercept)	1.974	0.095	20.766	** < 0.001**	25.64	400	** < 0.001**
March	0.325	0.105	3.093	** < 0.01**			
April	0.374	0.119	3.140	** < 0.01**			
May	0.735	0.139	5.366	** < 0.001**			
Sex: male	0.356	0.087	4.102	** < 0.001**			
Species: *F.* cf. *nicosiai*	− 1.022	0.103	− 9.921	** < 0.001**			

Bold indicates the significant values

**Table 2:** Parameters of the linear mixed model examining the influence of sex and time on H/L ratio in semi-captive *F. labordi*

Fixed effects	Estimate	SE	df	t-value	P	χ^2^	df	P
(Intercept)	1.185647	0.056175	163.3084	21.10641	** < 0.001**	33.75	5	** < 0.001**
sex: male	-0.00934	0.041367	33.16293	-0.22582	0.822			
March	0.222828	0.075443	239.0659	2.95359	** < 0.01**			
April	0.192228	0.079707	238.8112	2.411677	** < 0.05**			
May	0.39695	0,072,926	243.9702	5.443204	** < 0.001**			
June	0.295445	0.059703	175.842	4.948552	** < 0.001**			

Bold indicates the significant values

**Fig. 2:**
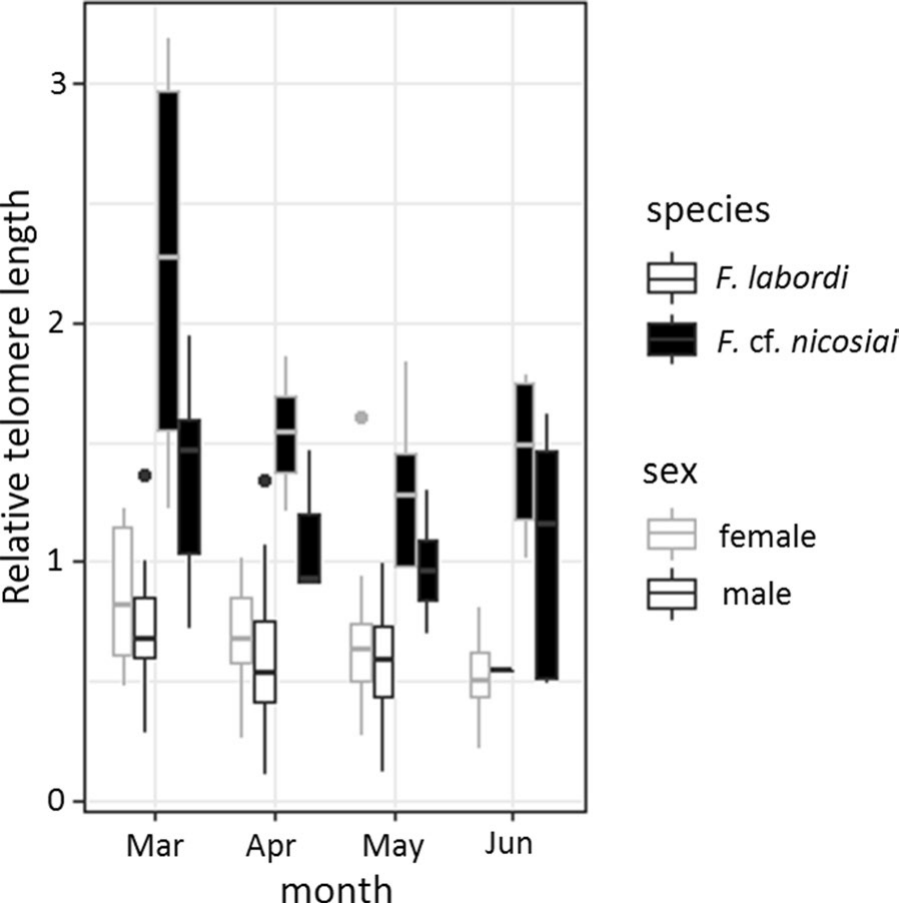
Relative telomere length of adult wild specimen of *F. labordi* (n = 66) and *F*. cf. *nicosiai* (n = 39). Boxplots depict relative TL per species and sex from March until June

**Fig. 3:**
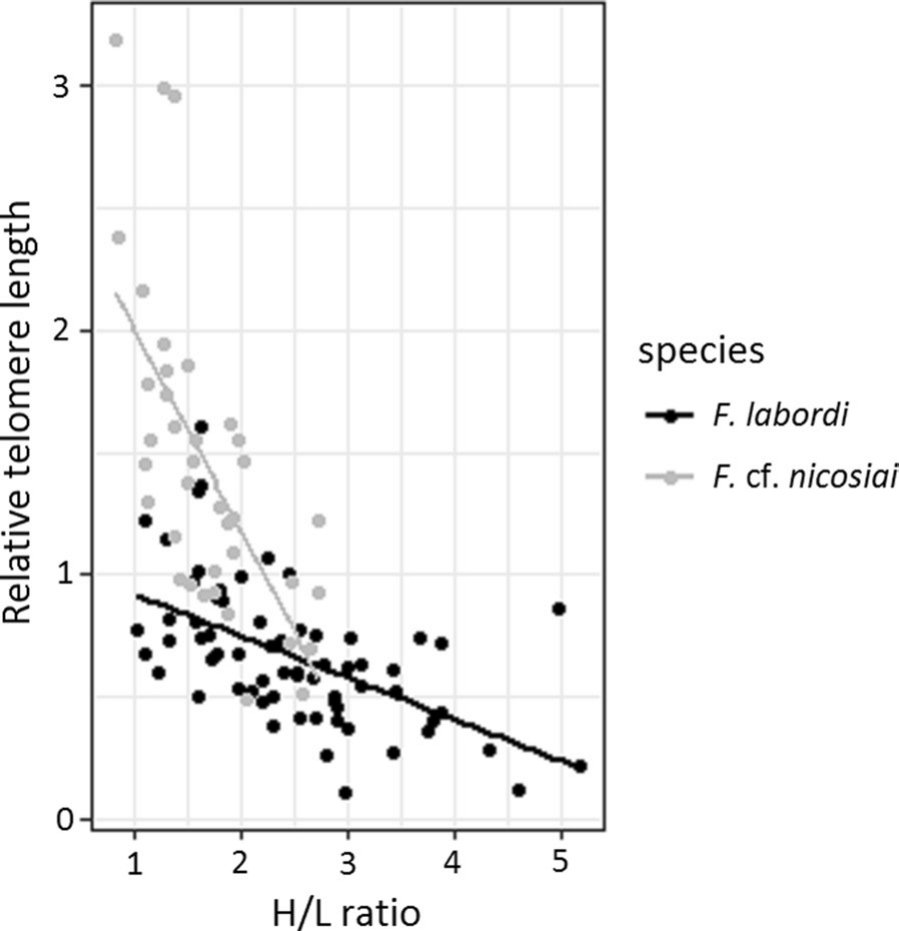
Relationship between H/L ratio and TL in *F. labordi* (n = 66) and *F*. cf. *nicosiai.* (n = 39)

During our sampling period, we did not detect any significant sex and age-related changes in TL in *F. labordi*. Average TL was significantly longer in *F.* cf. *nicosiai* (t = 6.438, p < 0.001). Furthermore, TL of *F*. cf. *nicosiai* was comparatively long in March (1.87 ± 0.77 SD, n = 14) and decreased dramatically until May (1.14 ± 0.33 SD, n = 10, t = − 2.686, p < 0.01). Moreover, TL of *F.* cf. *nicosiai* males was significantly shorter compared to females (t = − 2.67, p < 0.01, df = 38). For statistical analyses (Table 3), the months June and July were excluded due to small sample sizes (but June is included in Fig. 2), and we found a negative correlation between the H/L ratio and TL in *F. labordi* ($r$= − 0.556, df = 65, p < 0.01) and in *F.* cf. *nicosiai* ($r$ = − 0.687, df = 38, p $<0.01$; see Fig. 3).

**Table 3:** Parameters of the linear model examining the influence of time, sex and species on the telomere length of *F. labordi* and *F.* cf. *nicosiai*

Fixed effects	Estimate	SE	t-value	P	F	df	P
Intercept	0.8249	0.1162	7.100	** < 0.001**	25.67	80	** < 0.001**
Species *F.* cf. *nicosiai*	1.3070	0.1660	7.871	** < 0.001**			
April	− 0.1001	0.1291	− 0.775	0.441			
May	− 0.1343	0.1229	− 1.092	0.278			
sex male	− 0.1015	0.1055	− 0.962	0.339			
species F. cf. nicosiai April	− 0.3844	0.2378	− 1.616	0.11			
species *F*. cf. *nicosiai* May	− 0.5464	0.2001	− 2.731	** < 0.01**			
Species *F*. cf. nicosiai sex male	− 0.5176	0.1773	− 2.919	** < 0.01**			

Bold indicates the significant values

## Discussion

Our study revealed that H/L ratios were consistently higher in wild *F. labordi* compared to *F.* cf. *nicosiai,* hinting at higher stress levels in the shorter-lived species. *Furcifer labordi* already exhibited relatively short telomeres when they were 3–4 months old. TL was initially comparatively longer in *F*. cf. *nicosiai*, but undergoing rapid shortening after the mating season. In this species, we also detected intersexual differences in H/L ratio and TL, with shorter living males exhibiting higher H/L ratios and shorter telomeres. Interestingly, heterophils were the most common leucocyte type in both wild and captive chameleons. Captive *F. labordi* exhibited comparatively longer lifespans and lower H/L profiles than their wild conspecifics. In planning this study, we assumed that the captive chameleons would be buffered from some environmental stressors, like starvation, desiccation and predation risk. Our data therefore indicate that relatively long-lived wild *F. labordi* individuals were, on average, more stressed and lived shorter lives than their captive conspecifics, indicating a link between stress and longevity.

### Baseline stress levels and leukocyte profiles

Investigations in other reptile species indicated large differences between hematology values of different species as well as intraspecific variation as a function of season and sex [57, 58]. In their study of blood chemistry and hematology in captive panther chameleons (*Furcifer pardalis*), Laube et al. [59] found that lymphocytes were the predominant leukocyte type in both summer and winter. In contrast, Cuadrado et al. [60] reported that heterophils were the most frequently found leucocyte type in dystoic and healthy post-reproductive females of the common chameleon (*Chamaeleo chamaeleon*). The H/L ratio from that study (2.24) resembled the values reported here for *F. labordi* (2.45). More recently, Eshar et al. [61] found that heterophils were the most abundant leukocytes type in wild common chameleons. As part of their study of leukocyte profiles of an iguanid species, Davis et al. [62] reviewed several studies of white blood cell profiles of iguanids and other lizard species. They extracted data on the relative numbers of all cell types (mean percentages) and categorized the studies based on whether lizards were from captivity or the wild. They showed that all wild animals had higher H/L ratios than the captive conspecifics. In fact, the relative abundance of lymphocytes and heterophils was completely opposite in both groups, with lymphocytes being the most abundant leukocyte type in captive lizards and heterophils being most common one in wild specimens. Thus, either wild lizards naturally have higher baseline stress levels (and thus higher H/L ratios) than captive ones, or trapping of wild animals induced stress-related alterations in the animals’ leukocyte profiles, a notion also supported by the elevated H/L ratios of the captive *F. labordi* in our study.

During a stress response, GC secretion increases partly to mobilize more metabolic energy to deal with the stressor. While this stress response provides obvious short-term benefits, chronic elevation of GCs is harmful [19, 63–65]. In the present study, we observed stress-related changes in leukocyte profiles in both chameleon species, which may indirectly contribute to their rapid senescence after the reproductive season. Captive *F. labordi* showed comparatively lower, but in relation to other captive lizards, elevated H/L ratios [62], indicating that they perceived these captive conditions as mildly stressful, but that they were also buffered from major environmental stressors. It is possible that the brief biweekly handling to obtain blood samples might have contributed to the perceived stress level of caged individuals, but this manipulation did most likely not impact the measurements of H/L ratios because such effects were found only after 12 h in other species [25].

Any interpretation of the potential physiological effects of variable H/L ratios should take into account that a review published after our field work found inconsistent relationships between GC profiles and leukocyte profiles across studies [66]). In gopher tortoises, *Gopherus polyphemus*, both GC levels and leucocyte profiles changed across seasons, but the changes were not correlated [67]. Moreover, in two studies of garter snakes, *Thamnophis sirtalis,* conducted by the same research group, but on different populations and in different years, one study revealed a positive correlation between GC levels and H/L ratio [68], whereas the other did not [69]. Furthermore, the interpretation of leukocyte dynamics relies on baseline data for the taxon of interest [66]. Reports about leukocyte profiles in chameleons in the wild [60, 61] and captivity [59] are rare and based on relatively small sample sizes. Our study therefore contributes valuable comparative data based on large samples of two wild chameleon species, but future studies may want to assess stress levels more directly, e.g. by measuring GC levels from fecal samples.

### Telomere dynamics

Telomere dynamics differed between the two chameleon species. Telomeres were relatively longer in *F.* cf. *nicosiai,* but shortened rapidly with the disappearance of the adult cohort. In contrast, the telomeres of *F. labordi* were relatively short, but a deterioration over time was not detectable. The first 3 months in the life of *F. labordi* are characterized by fast growth rates, whereas juvenile *F.* cf. *nicosiai* show much slower growth and reach maturity at an age of 11–12 months [70]. The lifespan of *F.* cf. *nicosiai* is longer, but both species mate at the same time and die off afterwards. A study of wild jackdaws (*Corvus monedula)* revealed that long telomeres shorten more rapidly than short ones, regardless of the individual’s age [71]. Additionally, telomere degradation was highest in humans with long telomeres [72]. These studies suggest that mechanisms for telomere maintenance exist in vivo, which potentially protect the shortest telomeres from further attrition and might explain why we could not detect any significant TL reduction in *F. labordi*. It would therefore seem interesting to also examine telomerase activity in these species. In ectothermic vertebrates, the expression of telomerase is frequently found in somatic tissues and is thought to be due to the indeterminate growth [73]. Thus, regulation by this enzyme might enable *F. labordi* to maintain its TL up to a certain level.

Whether TL is a universal predictor of longevity is still up for debate. Whittemore et al. [74] found that the telomere shortening rate, but not the initial telomere length alone, is a powerful predictor of life span in several bird and mammal species. These results support the notion that critical telomere shortening and the consequent onset of telomeric DNA damage and cellular senescence are a general determinant of species life span. In humans, telomere attrition is also more rapid in the first decade of life, stabilizes in adulthood and is followed by a gradual loss at old age [75]. We could not study telomere dynamics because of low recapture rates and a lack of data on juveniles, but a relatively large male juvenile *F.* cf. *nicosiai* was sampled at approx. 4 months of age and showed a TL of 3.44, which was the highest measured in this species. In contrast, TL of hatchling pythons (*Lisais fuscus)* was significantly shorter than that of older snakes, increasing during their first year of life and subsequently decreasing with age [76]. Similar curvilinear telomere dynamics were found in frilled-necked lizards (*Chlamydosaurus kingii)* [77].

In *F*. cf. *nicosiai,* we also observed sexual dimorphism in telomere length across the sampling period, with females having longer average telomeres. The associated longer female survival may be adaptive as the maturation of eggs after insemination takes several weeks, and female chameleons are capable of producing additional clutches from stored sperm ([78], FE pers. observation). In several other species, including sand lizards (*Lacerta agilis)* [79], Medaka fish [80]) and humans [36], females also live longer and have longer telomeres. The actual mechanisms contributing to sex-specific telomere patterns are unknown, however. Previous work on humans suggested that the difference in TL stems from larger body mass in men compared to women [81], leading to the assumption that larger bodies require more tissue growth and cell division. However, female sand lizards are larger than males [82] and have longer telomeres. Gopalakrishnan et al. [80] postulated that estrogen is a key factor contributing to the decelerated telomere shortening in female Medaka fish, but corresponding data from other species are lacking. Thus, telomere attrition probably depends on multiple factors that remain to be identified.

Nowadays, telomere attrition is widely recognized as one of the hallmarks of aging (e.g. [83]), and telomeric assessments are widely used in evolutionary biology as biomarkers of somatic integrity. However, limited attention has been paid to addressing the fundamental question raised by these relationships: Which role do telomeres play in shaping the evolution of life history trade-offs and senescence [84]? While it is broadly accepted that telomere degradation can have causal effects on cell fates, the extent to which it contributes to age-related declines on organismal level is less clear. A proximate causal role for telomeres would more possibly reflect an adaptive strategy, born out of telomere maintenance costs and/or a function for telomere attrition (e.g. in counteracting cancer), the relative importance of which is currently unclear. Nevertheless, it is frequently mentioned that telomere length as a predictor of overall health could instead reflect it acting as a non-causal biomarker of accumulated damage to other biological structures that themselves have causal deleterious effects on the organismal performance (e.g. [85]). While it is mechanistically conceivable that telomere dynamics are one proximate cause of current–future trade-offs and senescence, whether telomeres play a significant proximate causal role relative to alternative mechanisms, such as oxidative damage to other biological structures, is currently uncertain [84]. Finally, advances in understanding of the selection pressures that might have shaped a proximate causal role for telomeres according to life history trade-offs have the potential to shed light on the nature of the evolutionary restrictions at play in life history evolution and help explain the form of the current–future trade-offs and ageing trajectories [84].

### Stress-related leukocyte profiles and telomere shortening

In both species, we found a negative correlation between average H/L ratio and TL. Chronical stress has potentially negative consequence through an increase in oxidative damage [42, 43] and ultimately telomere shortening [45]. Oxidative stress also dramatically decreases telomerase activity [86, 87]. Therefore, oxidative stress not only accelerates telomere shortening by direct damage to telomeres, but also by inhibiting telomere restoration as well. Even though we are well aware of the correlational nature of our study, we suggest that physiological stress negatively affected TL in our two study species. Although our findings and additional studies suggest a strong association between stress and telomere shortening [88, 89], we cannot discard other mechanisms that could affect TL, like alterations of early growth rates (e.g. [90]). More direct future studies should acknowledge that the link between stress response and telomere degradation is probably not straightforward and depends on the benefits and costs of activating an emergency life history state that is species- and context-dependent.

At an ultimate level, rates of extrinsic mortality are thought to determine where a species falls on the slow-fast continuum, with high rates of extrinsic mortality selecting for fast life histories [91]. The results of our previous capture-mark-recapture study [70] also suggest that extrinsic mortality rates in both chameleon species are presumably high in adults. Williams also postulated that juvenile mortality has no influence on the evolution of senescence; predicting that senescence should be associated with extrinsic mortality rates [100]. However, formal, mathematical theory [92–94] showed that this particular prediction is wrong. Accordingly, selection leading to senescence does not directly depend on survival to old age, but rather on the shape of the stable age distribution. The aim of evolutionary theories of aging is to clarify why organismal fitness mechanisms decline with age. Moorad et al. [95] therefore proposed to investigate the actual phenomenon of aging, not its proxies. More theory and careful physiological measurements from many species under many different environmental conditions are therefore required to further illuminate factors that shape life histories. Remarkably, Reznick and colleagues [96] even found that guppies (*Poecilia reticulata)* derived from natural populations with high levels of predation live the longest in the laboratory. This study demonstrates that our understanding of the evolution of senescence will profit from modeling numerous aging parameters, traits other than age at death as well as the causes of mortality.

Although there are many examples of negative correlations between lifespan and the apparent extrinsic risk of death faced by organisms, this risk is more often deduced than measured. In our study species, besides extrinsic mortality at old age, several factors might impact the short lifespan of this species. High juvenile mortality in *F. labordi* might lead to the extremely high investment in reproduction that in turn facilitates the pronounced stress response and relatively short telomeres. As physiological stress also has a strong influence on immune responses [97], the increasing gastrointestinal—and blood parasite burden observed in both species in the wild towards the dry season [98] might reflect an unavoidable consequence of this adaptation. This notion about the physiological processes contributing to such a short life span in *F. labordi* is also supported by a maximum lifespan in caged individuals of 16 months, indicating that their lifetime is indeed bounded by molecular and cellular mechanisms of aging.

## Conclusions

The results of our study provide rare information about leukocyte profiles and telomere dynamics in relation to senescence and mortality patterns of two chameleon species in the wild. The results of this study suggest that the presumably energetically demanding reproductive season in the short-lived species contributes to environmental stress ensued by increased oxidative damage and subsequent accelerated telomere shortening. To fully understand telomere dynamics and their relation to stress-related measures (H/L profiles) in these species, repeated samples from wild specimens and samples from younger life stages are necessary, however.

## Methods

### Study site and study species

This study was conducted in Kirindy Forest, which is located in the region of Menabe Central, Western Madagascar, ca. 60 km northeast of Morondava (44°39′E, 20°03′S, 30–60 m asl). It is one of the largest remaining Malagasy dry deciduous forest fragments. The local climate is characterized by a hot wet season between November and April, followed by a cool dry season from May to October [99]. Kirindy Forest is located near the northern end of the range of *Furcifer labordi*, a medium-sized and sexually highly dimorphic chameleon from the western and southwestern regions of Madagascar [96]. *Furcifer* cf. *nicosiai* is a relatively larger species, also sexually dimorphic [70], and appears to be limited to intact dry forests [100, 101].

### Capture-mark-recapture study

Wild chameleons were located at night using LED flashlights. The capture location was marked and GPS data were taken. We sampled alternating along two transects of 3 km length each. Animals were transported to the nearby research station in cloth bags and handled the following morning. They were sexed, age categorized (hatchling, juvenile, adult), and their snout vent length (SVL) was measured. Animals were individually marked by visual implant elastomers (VIE; Northwest Marine Technology Inc., Shaw Island, WA) [102]. Hatchlings and small juveniles were individually marked with nail polish on the toes. All chameleons were released at their point of capture within 12 h. Sampling took place over three field seasons: November 19, 2013–July 8, 2014, and January 11, 2015–July 15, 2015, and October 12, 2015–December 17, 2015.

### Experimental housing

We collected a total of 20 male and 20 female juveniles of *F. labordi* in early January, at approximately two months of age, and kept them individually without visual contact in cylindrical nylon cages (90 cm height, 60 cm diameter) inside a large outdoor forest enclosure. Chameleons received a standardized amount of insects, adjusted to their age and size to match growth and final size of the wild population. Specifically, animals were fed five times per week with two grasshoppers, crickets or butterflies. Water was offered daily with a spray flask. Between February and June, the caged animals were handled biweekly to obtain blood samples. Only captive animals were sampled repeatedly.

### Leukocyte profiles

To measure leukocyte profiles, a drop of blood was taken by lateral puncture of the caudal vein. No blood was taken from females that were obviously gravid. The drop of blood was placed on a microscope slide and distributed as a blood smear. After air-drying, blood smears were processed with a rapid differential haematology staining utilizing the Diff-Quik staining solution system (Medion Diagnostics AG, Düdingen, Switzerland). We determined the ratio of heterophils to lymphocytes (H/L ratio) in at least 200 cells per slide, using the 100 × oil objective. For identification, the general description of reptilian blood cells and terminology was used [103, 104]. Counting of the leukocytes started at the most distal edge of the feather end of the smear and proceeded one field of view at a time, across the entire smear in an ‘S’ fashion. Only fields of view with > 15 erythrocytes in a monolayer were considered [105]. All cell counting was conducted by FE. Blood samples were taken from February onwards until mid-July in 2014 and 2015. In total, 319 samples from wild *F. labordi*, 103 samples of *F.* cf. *nicosiai* and 278 samples from 40 captive animals were analysed.

### Telomere length (TL)

Blood samples for telomere analysis were taken between March and mid-July in 2015. In total, 66 blood samples of *F. labordi* and 39 of *F.* cf. *nicosiai* were obtained. Captive animals were excluded from this analysis because not enough blood samples were available.

For the determination of TL, we took approx. 5–10 µl blood from the caudal vein after lateral puncture with a capillary and transferred it into a 1.5 ml tube containing 0.5 ml SET buffer. Samples were directly frozen at − 20 °C. To avoid melting during transportation, samples were stored in a compressor cooling box. Telomere length was measured using real‐time quantitative PCR (qPCR) using SensiMix SYBR No‐ROX Kit (Bioline, Sydney, NSW, Australia) and a Rotor‐gene 6000 thermocycler (Qiagen, Chadstone, VIC, Australia) according to published protocols [106, 107] using techniques developed by [8] with the 18S ribosomal RNA (18S) gene as the non-variable copy number reference gene. The telomere primers used were Telb1 (5′‐CGGTTTGTTTGGGTTTGGGTTTGGGTTTGGGTTTGGGTT‐3′) and Telb2 (5′‐GGCTTGCCTTACCCTTACCCTTACCCTTACCCTTACCCT‐3′, (109)). The 18S gene (92 bp amplicon in Anolis) was selected as the reference gene as it had previously been validated in reptiles [8, 107, 109]. The primer sequences used were 18S‐F (5′‐GAGGTGAAATTCTTGGACCGG‐3′) and 18S‐R (5′‐CGAACCTCCGACTTTCGTTCT‐3′). Reactions were run in triplicate for each sample, with each run containing either Telb or 18S primers. Amplifications were carried out in a Rotor‐Gene 6000 thermocycler (Qiagen, Australia) using an initial Taq activation step at 95 °C for 10 min and a total of 40 cycles of 95 °C for 15 s, 60 °C for 15 s, and 72 °C for 15 s. Each reaction had a final volume of 20 μl with 10 ng of DNA. A melt curve was generated after each run over the temperature range of 60 to 95 °C to ensure that there was no nonspecific product amplification.

All of the DNA samples for a given individual were included in the same run. No‐template control reactions were run in triplicate for each primer set during every qPCR run to ensure that there was no contamination.

### Statistical analyses

Linear models (LM) were used to examine the influence of leukocyte profiles on TL in wild *F. labordi* and *F.* cf. *nicosiai*. As fixed factors, we added month (age), sex and species. For captive *F. labordi*, we used linear mixed models (LMM). As fixed factors, we added month (age), and sex, while ID was included as a random factor for recaptured samples. For all models, we compared the respective full model with the null model by using a likelihood ratio test. In addition, we visually inspected normality and homoscedasticity with residual plots. For model analysis, we used the package lme4 [109]. All data analysis was conducted in R (R-Code Team 2017) [110]. To check for correlation between H/L profile and TL, we calculated the Pearson correlation coefficient.

## Data Availability

All data generated or analyzed during this study are included in this published article [and its supplementary information files].

## Abbreviations

GC: Glucocorticoids
H/L ratio: Ratio heterophils:lymphocytes
TL: Telomere length
ROS: Reactive oxygen species

## References

[1] Stearns SC (1976). Life-history tactics: a review of the ideas. Q Rev Biol.

[2] Stearns SC. The evolution of life histories. Oxford Univ. 1992.

[3] Stearns SC (2000). Life history evolution: successes, limitations and prospects. Naturwissenschaften.

[4] Sæther BE, Bakke Ø (2000). Avian life history variation and contribution of demographic traits to the population growth rate. Ecology.

[5] Sæther BE, Engen S, Pape Møller A, Weimerskirch H, Visser ME, Fiedler W (2004). Life-history variation predicts the effects of demographic stochasticity on avian population dynamics. Am Nat.

[6] Salguero-Gómez R, Jones OR, Jongejans E, Blomberg SP, Hodgson DJ, Mbeau-Ache C (2016). Fast–slow continuum and reproductive strategies structure plant life-history variation worldwide. PNAS.

[7] Languille S, Blanc S, Blin O, Canale CI, Dal-Pan A, Devau G (2012). The grey mouse lemur: a non-human primate model for ageing studies. Aging Res Rev.

[8] Plot V, Criscuolo F, Zahn S, Georges JY (2012). Telomeres, age and reproduction in a long-lived reptile. PloS One..

[9] Barrett EL, Burke TA, Hammers M, Komdeur J, Richardson DS (2013). Telomere length and dynamics predict mortality in a wild longitudinal study. Mol Ecol.

[10] Fairlie J, Holland R, Pilkington JG, Pemberton JM, Harrington L, Nussey DH (2016). Lifelong leukocyte telomere dynamics and survival in a free-living mammal. Aging Cell.

[11] Sohal RS, Weindruch R (1996). Oxidative stress, caloric restriction, and aging. Science.

[12] Sena LA, Chandel NS (2012). Physiological roles of mitochondrial reactive oxygen species. Mol Cell.

[13] Salmon AB, Richardson A, Pérez VI (2010). Update on the oxidative stress theory of aging: does oxidative stress play a role in aging or healthy aging?. Free Radic Bio Med.

[14] Boonstra A, Barrat FJ, Crain C, Heath VL, Savelkoul HF, O’Garra A (2001). 1α, 25-Dihydroxyvitamin D3 has a direct effect on naive CD4+ T cells to enhance the development of Th2 cells. J Immunol.

[15] Ricklefs RE, Wikelski M (2002). The physiology/life-history nexus. Trends Ecol Evol.

[16] Romero LM, Reed JM, Wingfield JC (2000). Effects of weather on corticosterone responses in wild free-living passerine birds. Gen Comp Endocrinol.

[17] Sapolsky R, Rivier C, Yamamoto G, Plotsky P, Vale W (1987). Interleukin-1 stimulates the secretion of hypothalamic corticotropin-releasing factor. Science.

[18] Veldhuis JD, Sharma A, Roelfsema F (2013). Age-dependent and gender-dependent regulation of hypothalamic-adrenocorticotropic-adrenal axis. Endocrin Metab Clin.

[19] Sapolsky RM, Romero LM, Munck AU (2000). How do glucocorticoids influence stress responses? Integrating permissive, suppressive, stimulatory, and preparative actions. Endocr Rev.

[20] Dhabhar FS (2002). A hassle a day may keep the doctor away: stress and the augmentation of immune function. Integr Comp Biol.

[21] Bauer ME (2008). Chronic stress and immunosenescence: a review. Neuroimmunomodulat.

[22] Dallman MF (1993). Stress update: adaptation of the hypothalamic-pituitary-adrenal axis to chronic stress. Trend Endocrin Met.

[23] Wingfield JC, Romero LM. The endocrine system. In: McEwen BS, Goodman HM, editors. Handbook of physiology. 2001. p. 211–234.

[24] Romero LM, Reed JM (2005). Collecting baseline corticosterone samples in the field: is under 3 min good enough?. Comp Biochem Phys A.

[25] Davis AK, Maney DL, Maerz JC (2008). The use of leukocyte profiles to measure stress in vertebrates: a review for ecologists. Funct Ecol.

[26] Jain NC (1993). Essentials of veterinary hematology.

[27] Campbell TW (1995). Avian Hematology and Cytology.

[28] Rupley AE (1997). Manual of avian practice.

[29] Harmon BG (1998). Avian heterophils in inflammation and disease resistance. Poultry Sci.

[30] Thrall MA (2004). Hematology of amphibians, veterinary hematology and clinical chemistry: text and clinical case presentations.

[31] Campbell TW, Mader DR (1996). Clinical pathology. Reptile medicine and surgery.

[32] Meyne J, Ratliff RL, MoYzIs RK (1989). Conservation of the human telomere sequence (TTAGGG) n among vertebrates. PNAS.

[33] Prowse KR, Greider CW (1995). Developmental and tissue-specific regulation of mouse telomerase and telomere length. PNAS.

[34] McClintock B (1941). The stability of broken ends of chromosomes in *Zea mays*. Genetics.

[35] Watson JD (1972). Origin of concatemeric T7DNA. Nat New Biol.

[36] Gomes NM, Shay JW, Wright WE (2010). Telomere biology in metazoa. FEBS Lett.

[37] Stindl R (2004). Is telomere erosion a mechanism of species extinction?. J Exp Zool Part B.

[38] Eskes T, Haanen C (2007). Why do women live longer than men?. Eur J Obstet Gynecol Reprod Biol.

[39] Benetos A, Okuda K, Lajemi M, Kimura M, Thomas F, Skurnick J (2001). Telomere length as an indicator of biological aging: the gender effect and relation with pulse pressure and pulse wave velocity. Hypertension.

[40] Tanaka H, Horikawa I, Barrett JC, Oshimura M (2005). Evidence for inactivation of distinct telomerase repressor genes in different types of human cancers. Int J Cancer.

[41] Dantzer B, Fletcher QE (2015). Telomeres shorten more slowly in slow-aging wild animals than in fast-aging ones. Exp Gerontol.

[42] Agostinho P, Cunha RA, Oliveira C (2010). Neuroinflammation, oxidative stress and the patho-genesis of Alzheimer’s disease. Curr Pharm Des.

[43] Constantini D, Marasco V, Møller AP (2011). A meta-analysis of glucocorticoids as modulators of oxidative stress in vertebrates. J Comp Physiol B.

[44] Epel ES, Blackburn EH, Lin J, Dhabhar FS, Adler NE, Morrow JD, Cawthon RM (2004). Accelerated telomere shortening in response to life stress. PNAS.

[45] Wang Z, Rhee DB, Lu J, Bohr CT, Zhou F, Vallabhaneni H (2010). Characterization of oxidative guanine damage and repair in mammalian telomeres. PLoS Genet..

[46] Von Zglinicki T (2003). Replicative senescence and the art of counting. Exp Gerontol.

[47] Kotrschal A, Ilmonen P, Penn DJ (2007). Stress impacts telomere dynamics. Biol Lett.

[48] Haussmann MF, Marchetto NM (2010). Telomeres: linking stress and survival, ecology and evolution. Curr Zool.

[49] Shalev I, Moffitt TE, Sugden K, Williams B, Houts RM, Danese A (2013). Exposure to violence during childhood is associated with telomere erosion from 5 to 10 years of age: a longitudinal study. Mol Psychiatr.

[50] Bateson M (2016). Cumulative stress in research animals: telomere attrition as a biomarker in a welfare context?. BioEssays.

[51] Angelier F, Costantini D, Blevin P, Chastel O (2018). Do glucocorticoids mediate the link between environmental conditions and telomere dynamics in wild vertebrates? A review. Gen Comp Endocr.

[52] Houben JM, Moonen HJ, van Schooten FJ, Hageman GJ (2008). Telomere length assessment: biomarker of chronic oxidative stress?. Free Radic Biol Med.

[53] Karsten KB, Andriamandimbiarisoa LN, Fox SF, Raxworthy CJ (2008). A unique life history among tetrapods: an annual chameleon living mostly as an egg. PNAS.

[54] Eckhardt F, Kappeler PM, Kraus C (2017). Highly variable lifespan in an annual reptile, Labord’s chameleon (*Furcifer labordi*). Sci Rep.

[55] Braithwaite RW, Lee AK (1979). A mammalian example of semelparity. Am Nat.

[56] Oakwood M, Bradley AJ, Cockburn A (2001). Semelparity in a large marsupial. Proc R Soc Lond B Biol.

[57] Campbell W (2007). Ellis Avian and exotic animal hematology and cytology.

[58] Strik NI, Alleman R, Harr KE, Jacobson E (2007). Circulating inflammatory cells. Infectious diseases and pathology of reptiles, color atlas and text.

[59] Laube A, Pendl H, Clauss M, Altherr B, Hatt JM (2016). Plasma biochemistry and hematology reference values of captive panther chameleons (*Furcifer pardalis*) with special emphasis on seasonality and gender differences. J Zoo Wildl Med.

[60] Cuadrado M, Díaz-Paniagua C, Quevedo MA, Aguilar JM, Prescott IM (2002). Hematology and clinical chemistry in dystocic and healthy post-reproductive female chameleons. J Wildl Dis.

[61] Eshar D, Ammersbach M, Shacham B, Katzir G, Beaufrère H (2018). Venous blood gases, plasma biochemistry, and hematology of wild-caught common chameleons (*Chamaeleo chamaeleon*). Can J Vet Res.

[62] Davis AK, Ruyle LE, Maerz JC (2011). Effect of trapping method on leukocyte profiles of black-chested spiny-tailed iguanas (*Ctenosaura melanosterna*): implications for zoologists in the field. ISRN Zool..

[63] McEwen BS (2004). Protection and damage from acute and chronic stress: allostasis and allostatic overload and relevance to the pathophysiology of psychiatric disorders. Ann NY Acad Sci.

[64] Romero LM, Wikelski M (2001). Corticosterone levels predict survival probabilities of Galapagos marine iguanas during El Nino events. PNAS.

[65] Romero LM, Wikelski M (2002). Severe effects of low-level oil contamination on wildlife predicted by the corticosterone-stress response: preliminary data and a research agenda. Spill Sci Technol B.

[66] Davis AK, Maney DL (2018). The use of glucocorticoid hormones or leucocyte profiles to measure stress in vertebrates: what’s the difference?. Meth Ecol Evol.

[67] Goessling JM, Guyer C, Mendona MT (2016). Seasonal acclimation of constitutive immunity in gopher tortoises *Gopherus polyphemus*. Physiol Biochem Zool.

[68] Sparkman AM, Bronikowski AM, Williams S, Parsai S, Manhart W, Palacios MG (2014). Physiological indices of stress in wild and captive garter snakes: correlations, repeatability, and ecological variation. Comp Biochem Phys A.

[69] Gangloff EJ, Sparkman AM, Holden KG, Corwin CJ, Topf M, Bronikowski AM (2017). Geographic variation and within-individual correlations of physiological stress markers in a widespread reptile, the common garter snake (*Thamnophis sirtalis*). Comp Biochem Phys A.

[70] Eckhardt F, Kraus C, Kappeler PM (2019). Life histories, demographies and population dynamics of three sympatric chameleon species (*Furcifer* spp from western Madagascar. Amphibia-Reptilia..

[71] Salomons HM, Mulder GV, van de Zande L, Haussmann MF, Linskens MH, Verhulst S (2009). Telomere shortening and survival in free-living corvids. Proc R Soc B: Biol Sci.

[72] Nordfjäll K, Svenson U, Norrback KF, Adolfsson R, Lenner P, Roos G (2009). The individual blood cell telomere attrition rate is telomere length dependent. PLoS Genet..

[73] Gomes NM, Shay JW, Wright WE, Wolf NS (2010). Telomeres and telomerase. The comparative biology of aging.

[74] Whittemore K, Vera E, Martínez-Nevado E, Sanpera C, Blasco MA (2019). Telomere shortening rate predicts species life span. PNAS.

[75] Frenck RW, Blackburn EH, Shannon KM (1998). The rate of telomere sequence loss in human leukocytes varies with age. PNAS.

[76] Ujvari B, Madsen T (2009). Short telomeres in hatchling snakes: erythrocyte telomere dynamics and longevity in tropical pythons. PloS One..

[77] Ujvari B, Biro PA, Charters JE, Brown G, Heasman K, Beckmann C, Madsen T (2017). Curvilinear telomere length dynamics in a squamate reptile. Funct Ecol.

[78] Tolley KA, Chauke LF, Jackson JC, Feldheim KA (2014). Multiple paternity and sperm storage in the Cape dwarf chameleon (*Bradypodion pumilum*). Afr J Herpetol.

[79] Olsson M, Pauliny A, Wapstra E, Uller T, Schwartz T, Miller E, Blomqvist D (2011). Sexual differences in telomere selection in the wild. Mol Ecol.

[80] Gopalakrishnan S, Cheung NK, Yip BW, Au DW (2013). Medaka fish exhibits longevity gender gap, a natural drop in estrogen and telomere shortening during aging: a unique model for studying sex-dependent longevity. Front Zool.

[81] Seluanov A, Chen Z, Hine C, Sasahara TH, Ribeiro AA, Catania KC (2007). Telomerase activity coevolves with body mass not lifespan. Aging Cell.

[82] Gullberg A, Olsson M, Tegelström H (1997). Male mating success, reproductive success and multiple paternity in a natural population of sand lizards: behavioural and molecular genetics data. Mol Ecol.

[83] Lopez-Otin C, Blasco MA, Partridge L, Serrano M, Kroemer G (2013). The hallmarks of aging. Cell.

[84] Young AJ (2018). The role of telomeres in the mechanisms and evolution of life-history trade-offs and ageing. Philos Trans R Soc B.

[85] Simons MJP (2015). Questioning causal involvement of telomeres in aging. Ageing Res Rev.

[86] Borrás C, Esteve JM, Viña JR, Sastre J, Viña J, Pallardó FV (2004). Glutathione regulates telomerase activity in 3T3 fibroblasts. J Biol Chem.

[87] Kurz DJ, Decary S, Hong Y, Trivier E, Akhmedov A, Erusalimsky JD (2004). Chronic oxidative stress compromises telomere integrity and accelerates the onset of senescence in human endothelial cells. J Cell Sci.

[88] Haussmann MF, Longenecker AS, Marchetto NM, Juliano SA, Bowden RM (2011). Embryonic exposure to corticosterone modifies the juvenile stress response, oxidative stress and telomere length. Proc R Soc B: Biol Sci.

[89] Quirici V, Guerrero CJ, Krause JS, Wingfield JC, Vásquez RA (2016). The relationship of telomere length to baseline corticosterone levels in nestlings of an altricial passerine bird in natural populations. Front Zool.

[90] Schultner J, Moe B, Chastel O, Bech C, Kitaysky AS (2014). Migration and stress during reproduction govern telomere dynamics in a seabird. Biol Lett.

[91] Williams GC (1957). Pleiotropy, natural selection, and the evolution of senescence. Evolution.

[92] Abrams PA (1993). Does increased mortality favor the evolution of more rapid senescence?. Evolution.

[93] Caswell H (2007). Extrinsic mortality and the evolution of senescence. Trend Ecol Evol.

[94] Wensink MJ, Caswell H, Baudisch A (2017). The rarity of survival to old age does not drive the evolution of senescence. Evol Biol.

[95] Moorad J, Promislow D, Silvertown J (2019). Evolutionary ecology of senescence and a reassessment of Williams’ ‘Extrinsic Mortality’ Hypothesis. Trend Ecol Evol..

[96] Reznick DN, Bryant MJ, Roff D, Ghalambor CK, Ghalambor DE (2004). Effect of extrinsic mortality on the evolution of senescence in guppies. Nature.

[97] Padgett DA, Glaser R (2003). How stress influences the immune response. Trend Immunol.

[98] Eckhardt F, Strube C, Mathes KA, Mutschmann F, Thiesler H, Kraus C, Kappeler PM (2019). Parasite burden in a short-lived chameleon, *Furcifer labordi*. Int J Parasitol PAR.

[99] Kappeler PM, Fichtel C. A 15-year perspective on the social organization and life history of sifaka in Kirindy Forest. In: Long-term field studies of primates. Berlin: Springer. 2012. p. 101–121

[100] Glaw F, Vences M. A field guide to the amphibians and reptiles of Madagascar. Vences & Glaw. 2007.

[101] Jesu R, Mattioli FSG (1999). On the discovery of a new large chameleon inhabiting the limestone outcrops of western Madagascar: *Furcifer nicosiai* sp. nov. (Reptilia, Chamaeleonidae). Doriana..

[102] MacNeil JE, Dharmarajan GUHA, Williams RN (2011). Salamarker: a code generator and standardized marking system for use with visible implant elastomers. Herpetol Conserv Bio.

[103] Origgi FC, Jacobson E (2007). Reptile immunology. Infectious diseases and pathology of reptiles, color atlas and text.

[104] Tamukai K, Takami Y, Akabane Y, Kanazawa Y, Une Y (2011). Plasma biochemical reference values in clinically healthy captive bearded dragons (*Pogona vitticeps*) and the effects of sex and season. Vet Clin Path.

[105] Davis AK, Maerz JC (2008). Comparison of hematological stress indicators in recently captured and captive paedomorphic mole salamanders, *Ambystoma talpoideum*. Copeia.

[106] Rollings N, Friesen CR, Sudyka J, Whittington C, Giraudeau M, Wilson M, Olsson M (2017). Telomere dynamics in a lizard with morph-specific reproductive investment and self-maintenance. Ecol Evol.

[107] Rollings N, Uhrig EJ, Krohmer RW, Waye HL, Mason RT, Olsson M (2017). Age-related sex differences in body condition and telomere dynamics of red-sided garter snakes. Proc R Soc Lond Ser B Biol Sci..

[108] Cawthon RM (2002). Telomere measurement by quantitative PCR. Nucleic Acids Res.

[109] Rollings N, Friesen CR, Whittington CM, Johansson R, Shine R, Olsson M (2019). Sex- and tissuespecific differences in telomere length in a reptile. Ecol Evol.

[110] Team RC. R: a language and environment for statistical computing. R Foundation for Statistical Computing, Vienna, Austria. https://www.R-project.org. 2017.

